# Supplementary material for: Charging and ultralong phosphorescence of lanthanide facilitated organic complex
Source: Nat Commun. 2021 Nov 11;12:6532. doi: 10.1038/s41467-021-26927-z (PMC8586359; doi:10.1038/s41467-021-26927-z)
Supplement: Supplementary file 1 — Supplementary Information [file 41467_2021_26927_MOESM1_ESM.pdf]

Supplementary Information

Charging and Ultralong Phosphorescence of Lanthanide Facilitated Organic Complex

Waygen Thor[1], Yue Wu[1], Lei Wang[1], Yonghong Zhang[1,2], Peter A. Tanner[1*], Ka-Leung Wong[1*]

[1]Department of Chemistry, Hong Kong Baptist University, Waterloo Road, Kowloon Tong, Hong Kong S.A.R. [2]State Key Laboratory of Chemistry and Utilization of Carbon Based Energy Resources, Key Laboratory of Oil and Gas Fine Chemicals, Ministry of Education & Xinjiang Uygur Autonomous Region, Urumqi Key Laboratory of Green Catalysis and Synthesis Technology, College of Chemistry, Xinjiang University, Urumqi 830046, Xinjiang, PR China.

	Title	Page, S
Supplementary Fig. 1	Chemical structure of **LaPhen(TTA)$_3$**	1
Supplementary Fig. 2	NMR Spectra of **L1** and **LaL1(TTA)$_3$**	2
Supplementary Fig. 3	Room temperature photophysical properties of **LaL1(TTA)$_3$**	3
Supplementary Fig. 4	Photophysical properties of **LaL1(TTA)$_3$** at 77 K	4
Supplementary Fig. 5	Photophysical properties of **LaL1(TTA)$_3$** at different temperatures	5
Supplementary Fig. 6	Photophysical properties of **L1**	6
Supplementary Fig. 7	Computational studies of **LaL1(TTA)$_3$** and **L1**	7
Supplementary Fig. 8	Structural conformation of **L1** dimer	7
Supplementary Fig. 9	Time resolved intensity profile of **LaL1(TTA)$_3$**	8
Supplementary Fig. 10	Kinetics Simulation of **LaL1(TTA)$_3$** with time	8
Supplementary Fig. 11	Proposed mechanism for photophysical processes of **LaL1(TTA)$_3$**	9
Supplementary Fig. 12	Phase stability of **LaL1(TTA)$_3$**	10

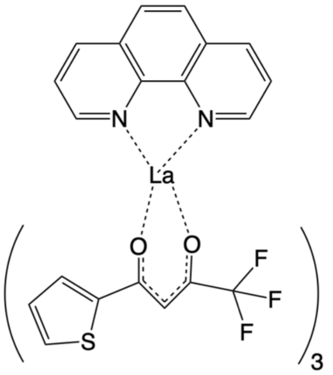

LaPhen(TTA)$_3$

Supplementary Fig. 1 | Chemical structure. The chemical structure of **LaPhen(TTA)$_3$** used as a comparison in this work.

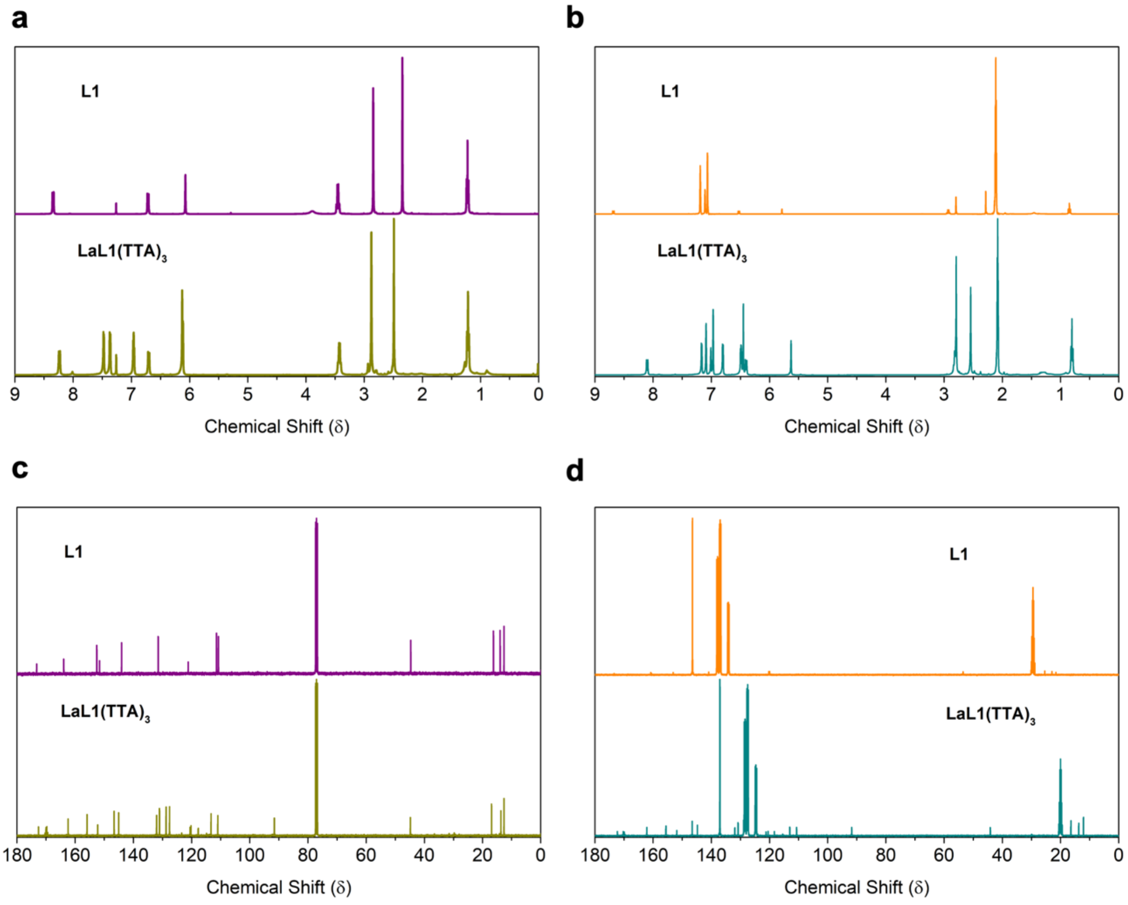

Supplementary Fig. 2 | NMR spectra. The ^{1}H NMR of **L1** and **LaL1(TTA)$_3$** in (**a**) $CDCl_3$, (**b**) toluene-d_8 and the ^{13}C NMR in (**c**) $CDCl_3$, (**d**) toluene-d_8 recorded using 400 MHz NMR spectrometer. The good agreement and minor shifting of the peaks between complex and ligands at high concentration in the NMR spectra show that the ligands are coordinated to the metal.

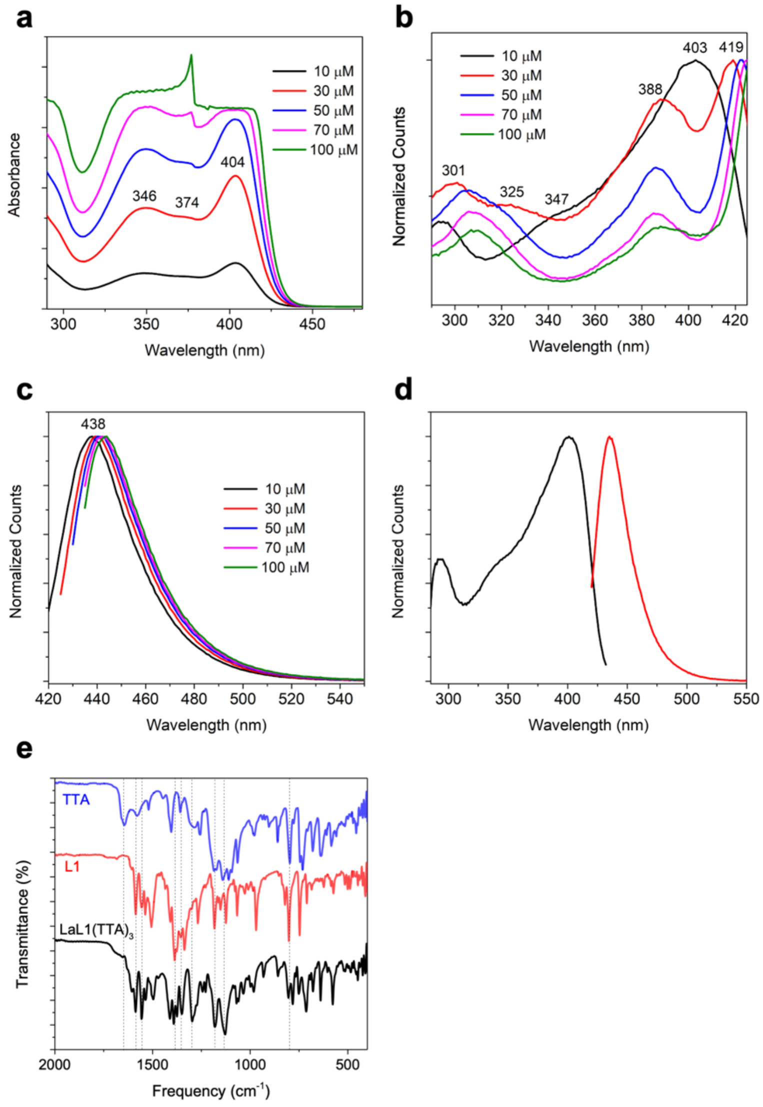

Supplementary Fig. 3 | Room temperature photophysical properties of LaL1(TTA)$_3$. (a) Absorption spectra (there is a grating change at 377 nm). **(b)** Excitation spectra taken at emission band maxima. The inner filter effect is responsible for the "dip" at 403 nm with increasing concentration. The bands broaden with increasing concentration. **(c)** Emission spectra taken at excitation band maxima at different concentrations. **(d)** Overlay of 10 μM excitation (λ_{em} = 435 nm; black) and emission (λ_{exc} = 400 nm; red) spectra. (**e**) Comparison of FT-IR spectra of **TTA**, **L1** and **LaL1(TTA)$_3$**.

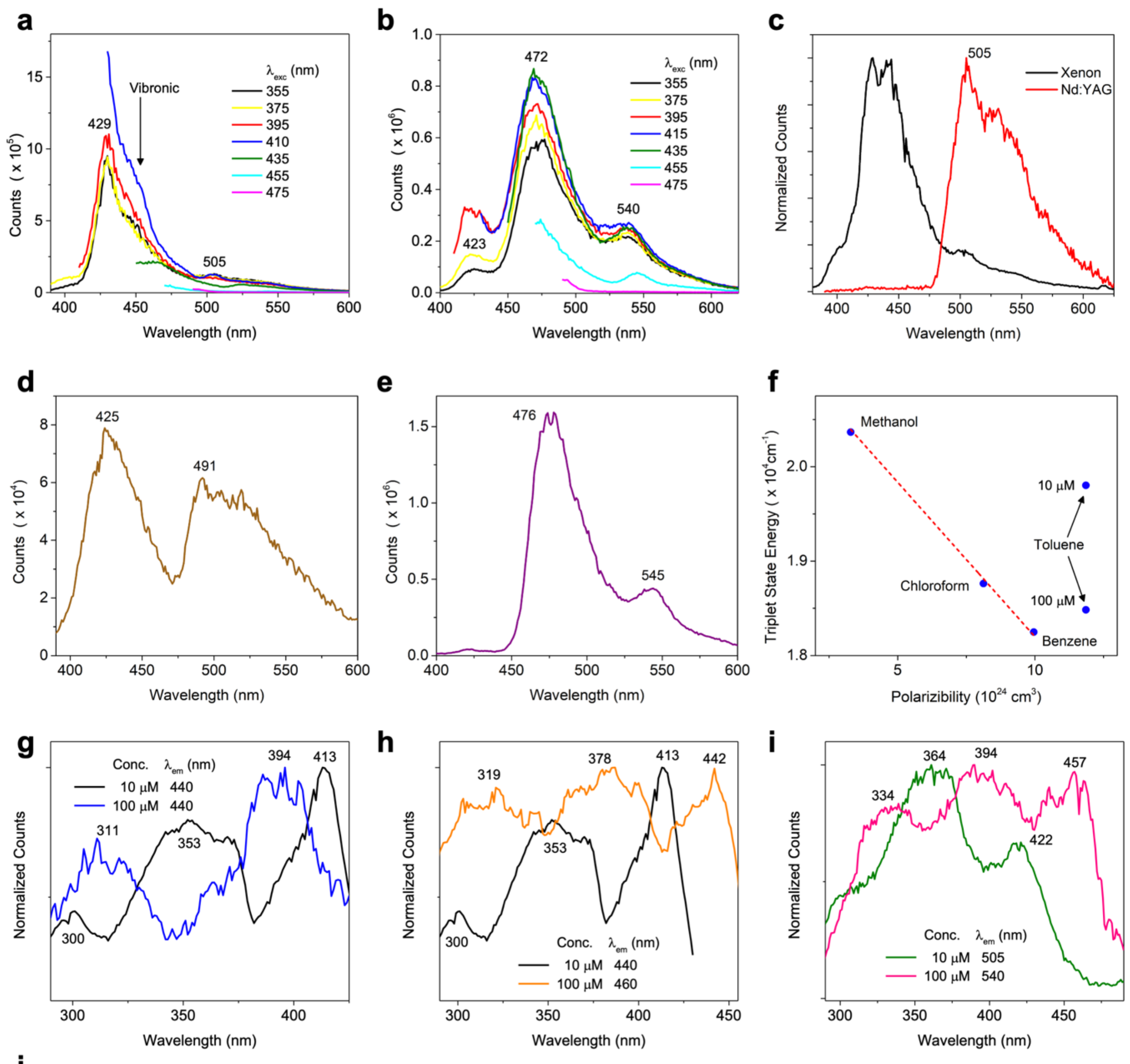

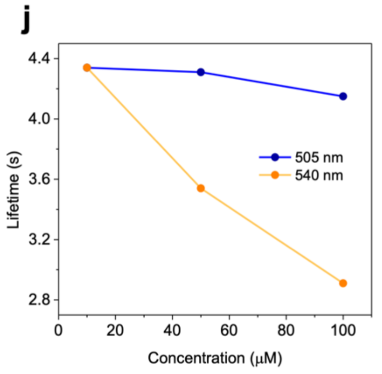

k

Relative Intensity	Lifetime (s) Charging	Lifetime (s) Decay
**100**	2.81 ± 0.11	4.32 ± 0.11
**54**	3.52 ± 0.11	4.28 ± 0.04
**33**	3.93 ± 0.13	4.35 ± 0.09
**11**	4.29 ± 0.18	4.36 ± 0.12

Supplementary Fig. 4 | Photophysical properties of LaL1(TTA)$_3$ at 77 K. Emission spectra of **LaL1(TTA)$_3$** in (**a**) toluene (10 μM) and (**b**) $CHCl_3$ (10 μM) with varying excitation wavelength (in nm). (**c**) Emission spectra of **LaL1(TTA)$_3$** in toluene (10 μM) with different excitation source and photomultiplier tube detection: continuous wave Xe lamp λ_{exc} = 355 nm (black) and 10 Hz pulsed Nd^{3+}:YAG laser at 355 nm (red). The emission spectrum of **LaL1(TTA)$_3$** at 10 μM concentration in (**d**) methanol and in (**e**) benzene, with the singlet and triplet energy maxima labeled. (**f**) The plot of triplet state energy maximum versus solvent polarizability shows a linear relation for three solvents. The deviation for the **LaL1(TTA)$_3$** triplet state energy at 100 μM concentration in toluene from the straight line is attributed to the steric factor and especially, the electron-donating ability of the CH_3 group substitution in benzene. π-stacking interactions are highly-dependent upon geometry. The excitation

spectra for **LaL1(TTA)₃** in toluene with 10 μM and 100 μM concentrations at (**g**) λ_{em} = 440 nm, (**h**) emission maximum of the respective singlet state, and (**i**) of the respective triplet state. The difference in the excitation spectra shows that a different structure is present in the electronic ground state of the high and low concentration forms of **LaL1(TTA)₃**. (**j**) The phosphorescence decay lifetime of **LaL1(TTA)₃** monitored at different concentrations, showing the decrease when form B dominates. (**k**) The charging (rise) and decay lifetimes averaged over 5 cycles of **LaL1(TTA)₃** with different neutral density (ND) filters in the excitation beam. The higher the excitation power, the bigger the difference between the rise lifetime and decay lifetime.

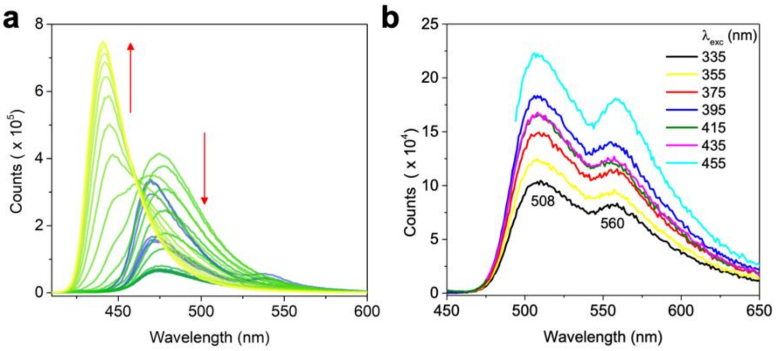

c

Temperature (K)	10	50	100	150
τ (s)	2.06 ± 0.02	1.65 ± 0.03	1.18 ± 0.03	0.46 ± 0.05

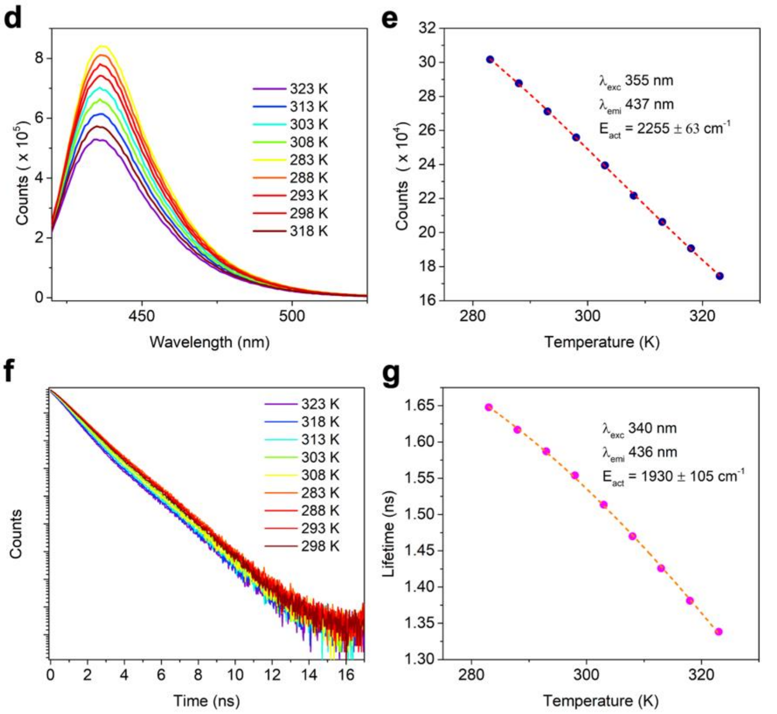

Supplementary Fig. 5 | Photophysical properties of LaL1(TTA)₃ at different temperatures. (**a**) The changes in the emission spectra of 100 μM **LaL1(TTA)₃** in toluene recorded gradually when heating up from 77 K to room temperature. The temperature-dependent emission spectra demonstrate that both species (form A and B) coexist, with the aggregated form B dominating at high concentration. (**b**) Emission spectra of solid **LaL1(TTA)₃** at ~100 K with varying excitation wavelength. (**c**) Variation of the phosphorescence decay lifetime of solid **LaL1(TTA)₃** (λ_{exc} = 355 nm, λ_{em} = 560 nm) with temperature. (**d**) The singlet emission intensity of **LaL1(TTA)₃** decreases with increasing temperature, which demonstrates (**e**) an activation energy with 2255 ± 63 cm^{-1}. Similarly, the (**f**) lifetime decreases with increasing temperature with an (**g**) activation energy of 1930 ± 105 cm^{-1}.

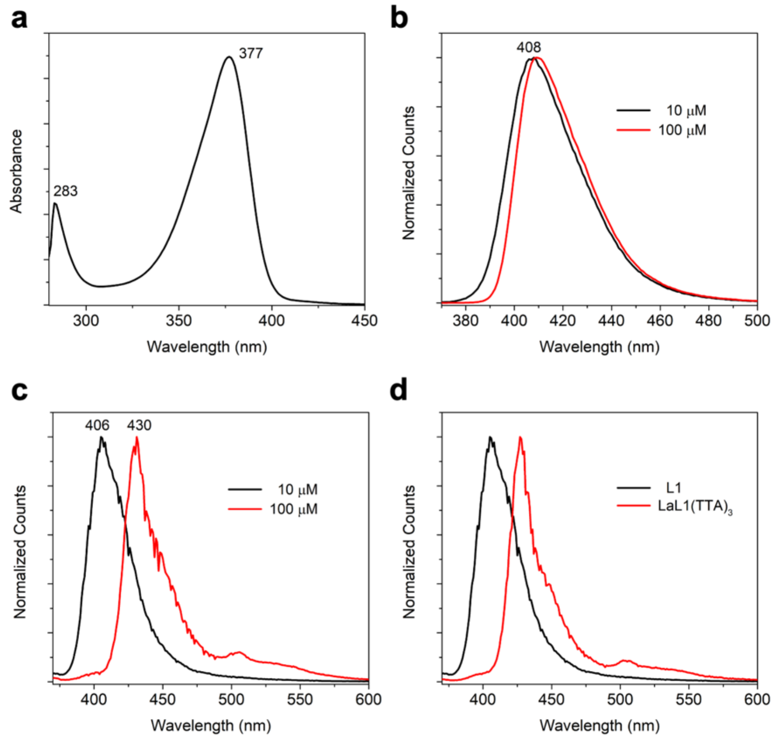

Supplementary Fig. 6 | Photophysical properties of L1. (**a**) Absorption spectrum of free ligand **L1** (10 μM in toluene). The band is shifted 25 nm to low energy in the complex. (**b**) Concentration dependence of room temperature emission spectrum. (**c**) Concentration dependence of 77 K emission spectrum. (**d**) Comparison of **L1** and **LaL1** emission spectra (λ_{exc} = 355 nm, T = 77 K, concentration 10 μM).

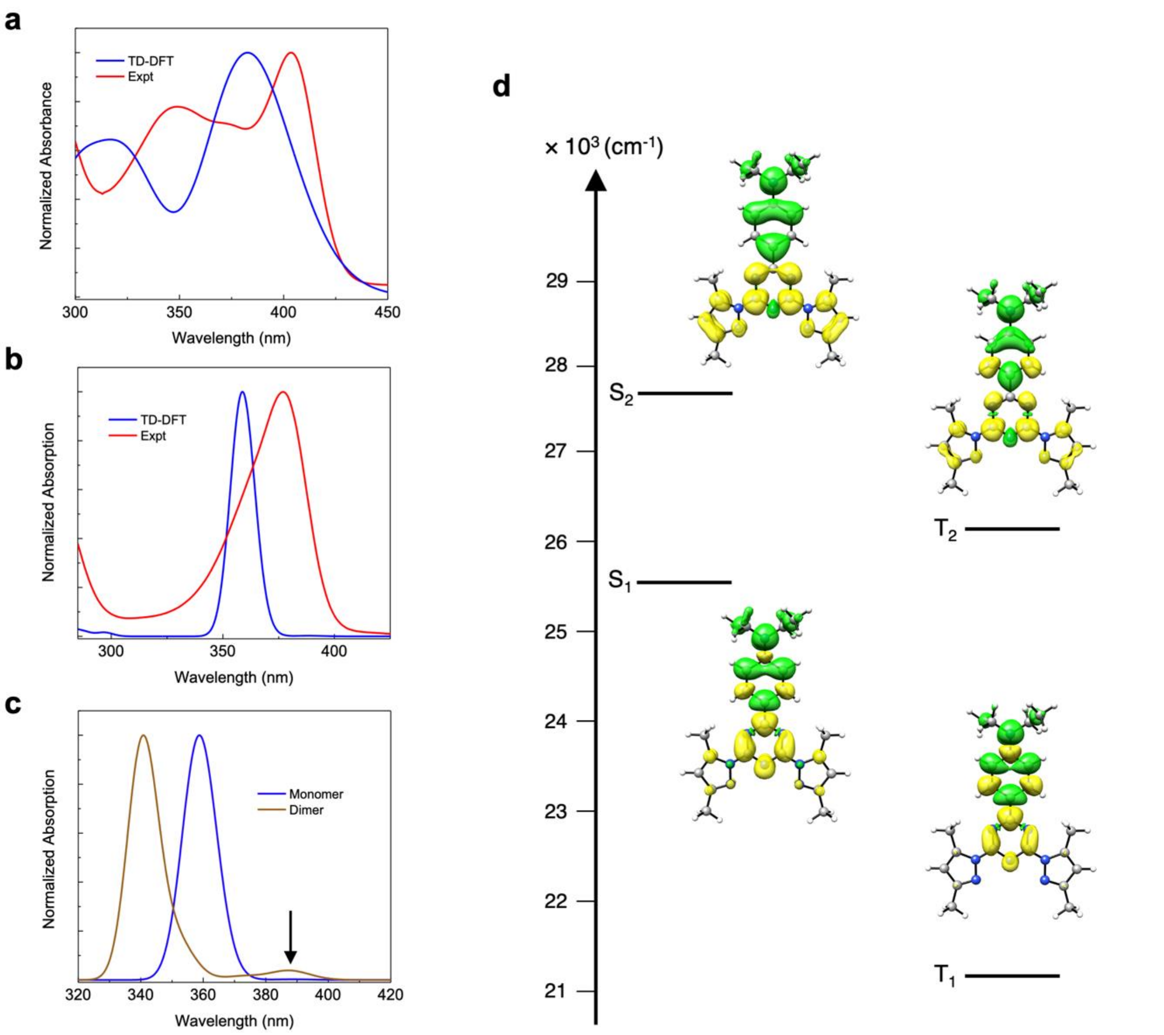

Supplementary Fig. 7 | Computational studies of LaL1(TTA)$_3$ and L1. The comparison of experimental absorption spectra at 10 μM concentration with TD-DFT calculation for (**a**) **LaL1(TTA)$_3$** and (**b**) **L1**. The calculated monomer-dimer shift is evident in (**c**) with the arrow. (**d**) The orbital transitions for the lowest singlet and triplet transitions of **L1**. The green colour of the orbitals represents the decrease in occupation during the electronic transition whilst yellow represents an increase. The lowest singlet and triplet transitions are of intra-ligand charge character.

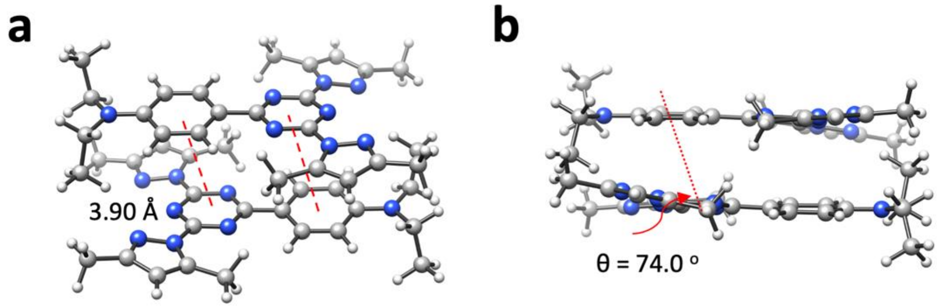

Supplementary Fig. 8 | Structural conformation of L1 dimer. The (**a**) lateral view and (**b**) the side view of the optimized structure of **L1** dimer. The distance between the benzene ring and the triazine ring of the neighbouring **L1** is 3.90 Å with a dihedral angle of 74.0°.

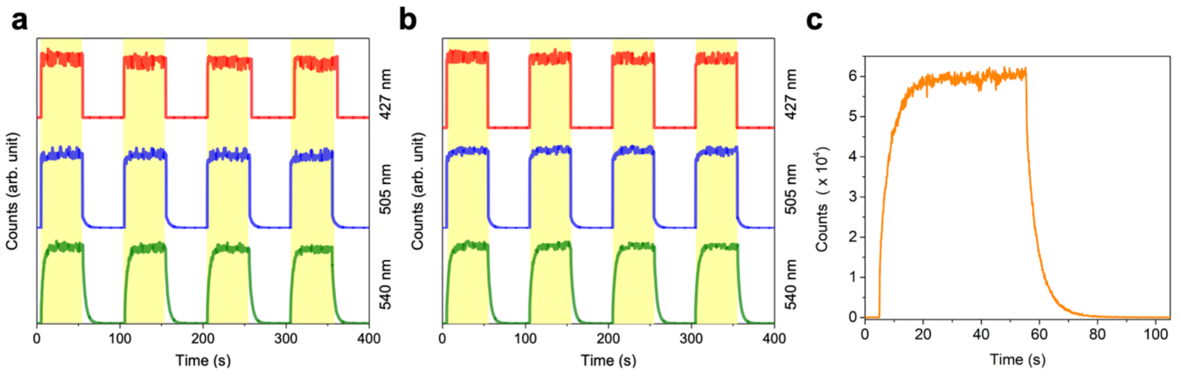

Supplementary Fig. 9 | Time resolved intensity profile of LaL1(TTA)₃. The time resolved intensity profile of 100 μM **LaL1(TTA)₃** in toluene with (**a**) λ_{exc} = 355 nm and (**b**) λ_{exc} = 395 nm at 77 K, monitoring at the wavelengths indicated on the right hand side. The enlarged charging and decay profile for (**c**) 10 μM **LaL1(TTA)₃** at 77 K (λ_{exc} = 355 nm, λ_{em} = 505 nm).

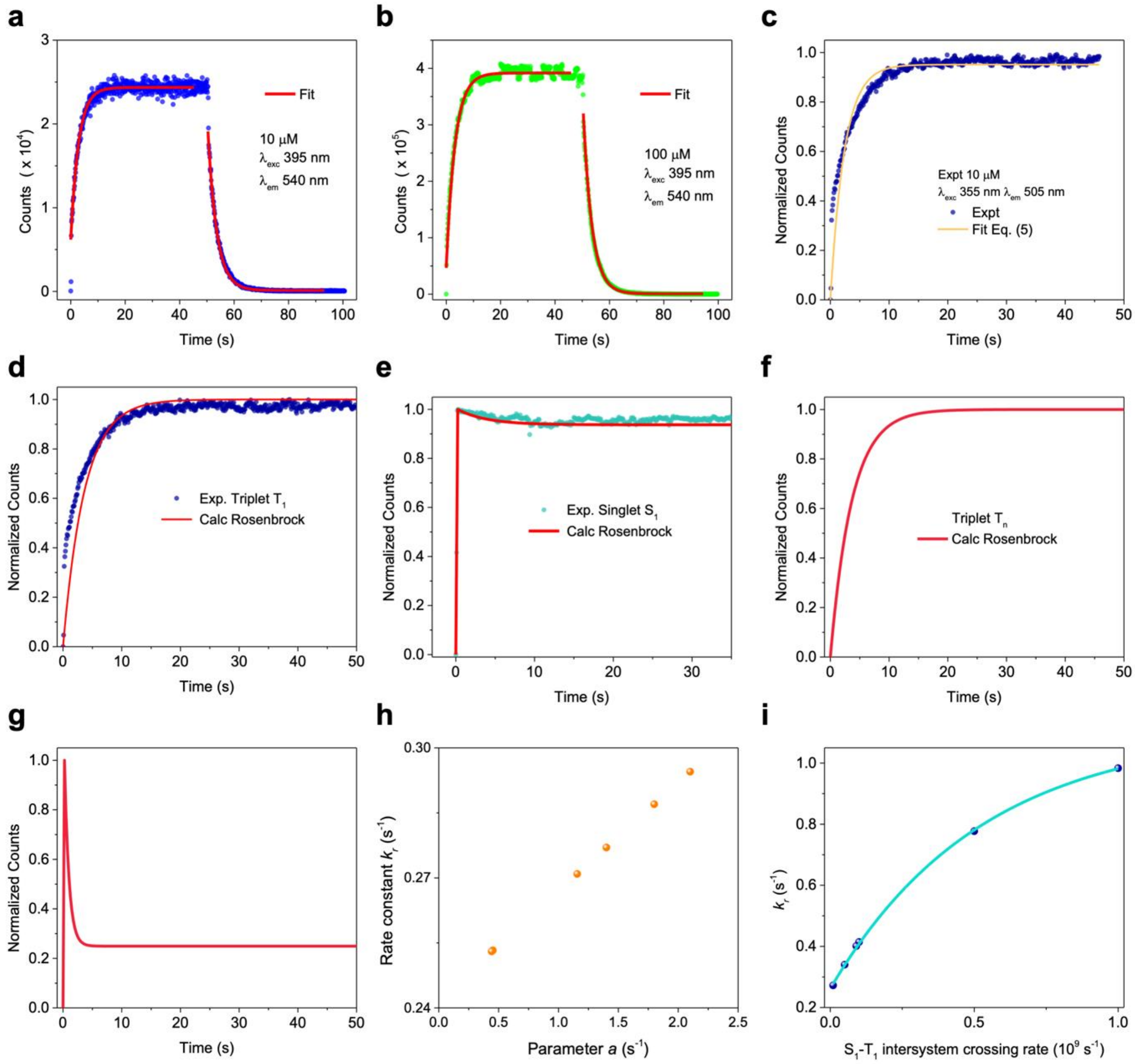

Supplementary Fig. 10 | Kinetics Simulation of LaL1(TTA)₃ with time. (**a**), (**b**) Monoexponential fits to the rise and decay of 10 μM and 100 μM **LaL1(TTA)₃** in toluene at 77 K under the conditions indicated. The emission

occurs from the triplet state of form A (**a**) and form B (**b**). (**c**) The fit of Eq. (5) to the triplet rise for 10 μM **LaL1(TTA)₃** in toluene at 77 K (λ_{exc} = 355 nm, λ_{em} = 505 nm). (**d**),(**e**) Use of Rosenbrock stiff 3-4th order numerical method for solution of charging profiles using the rate equations (1)-(4). The modified extended backward differentiation equation implicit method gives similar results. The simulations are not data fits and employ the fixed arbitrary parameter set (in s^{-1}): a = 1.215, b = 6.5E8, c = 1E7, d = 0.242, f = 1.215, g = 1E10, h = 1E8, k = 1E6. The initial concentrations (t = 0) are set at [S_0] = 1, [S_1] = 0, [T_1] = 0, [T_n] = 0. More detailed calculations have been carried out which distinguish radiative and nonradiative rates for S_1 and T_1 as well as the excited state absorption of S_1 but they do not add further to our understanding. The experimental data are taken from 10 μM **LaL1(TTA)₃** in toluene at 77 K (λ_{exc} = 355 nm, λ_{em} = 427 nm for S_1 and 505 nm for T_1). The decrease in (**e**) following the initial sharp rise is scarcely observable for the mainly monomeric species but is captured in the simulation. (**f**) Calculated charging time profile for T_n using the same parameter set. (**g**) Effect upon the S_1 charging profile of changing the intersystem crossing rate parameter c alone from 1E7 in (**e**) to 1E9. (**h**) Calculated variation of triplet rise rate constant, k_r, with parameter a (which is proportional to incident intensity). Parameter f is set to the same value as a. Increasing parameter f alone in the above set (for example, from 1E10 to 1E12) increases the value of k_r. (**i**) Calculated T_1 (charging) rise rate constant by varying parameter c only in the above parameter set.

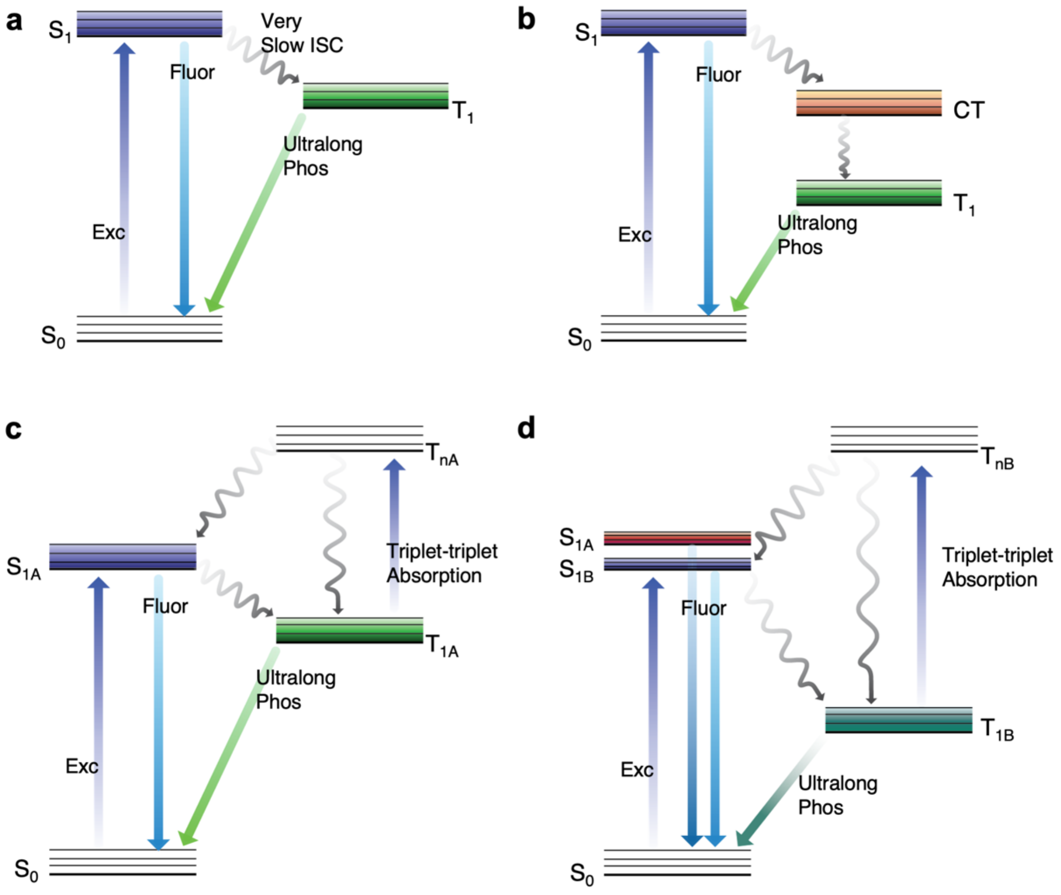

Supplementary Fig. 11 | Proposed mechanism for photophysical processes of LaL1(TTA)₃. The other proposed mechanisms where (**a**) there is a slow intersystem crossing rate from S_1 to T_1 and (**b**) the existence of a charge-transfer state between S_1 and T_1 are both dismissed. Suggestion (**a**) would not account for the difference in rise and decay constants of T_1. There is no spectroscopic evidence for (**b**), a charge transfer state. (**c**),(**d**) The triplet-triplet absorption proposed for **LaL1(TTA)₃**, while that for (**d**) is the case for **LaL1(TTA)₃** of higher concentration with the presence of the stacked conformation.

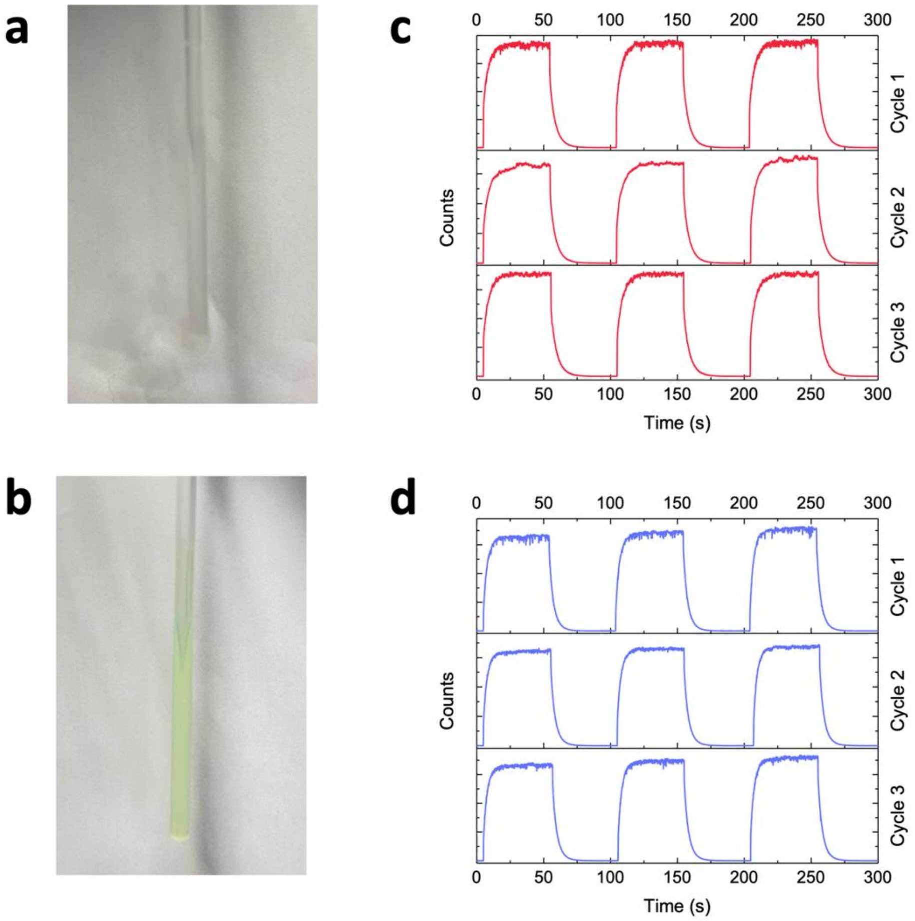

Supplementary Fig. 12 | Phase stability of LaL1(TTA)$_3$. The photo of **LaL1(TTA)$_3$** in (**a**) 10 μM and (**b**) 100 μM toluene under cryogenic condition demonstrate a clear single liquid crystal phase, demonstrating good phase stability upon cooling. The repeated cooling-heating process of **LaL1(TTA)$_3$** in (**c**) 10 μM (λ_{exc} = 355 nm, λ_{em} = 505 nm) and (**d**) 100 μM toluene (λ_{exc} = 355 nm, λ_{em} = 540 nm) shows similar optical processes, indicating good stability upon repeated measurements.
