# Supplementary material for: Charging and ultralong phosphorescence of lanthanide facilitated organic complex
Source: Nat Commun. 2021 Nov 11;12:6532. doi: 10.1038/s41467-021-26927-z (PMC8586359; doi:10.1038/s41467-021-26927-z)
Supplement: Supplementary file 3 — Description of Additional Supplementary Files [file 41467_2021_26927_MOESM3_ESM.pdf]

Description of Additional Supplementary Files

Supplementary Movie 1: Upon irradiation at 77K, 10 μM **LaL1(TTA)$_3$** in toluene shows a bright green afterglow which was observable up to 30 s by the naked eye under 355 nm laser excitation.

Supplementary Movie 2: Under the same conditions, the afterglow of **L1** is observed above 5 s.

Supplementary Movie 3: Long-lived phosphorescence can also be seen under white light excitation for **LaL1(TTA)$_3$** at 100 μM concentration.

Supplementary Movie 4: The vessel shows blue emission from all squares at room temperature.

Supplementary Movie 5: However, the desirable logo can be captured after excitation with a UV lamp under the cryogenic state.
